# Hypothyroidism and Its Impact on Menstrual Irregularities in Reproductive-Age Women: A Comprehensive Analysis at a Tertiary Care Center

**DOI:** 10.7759/cureus.63158

**Published:** 2024-06-25

**Authors:** Hannah Himabindu P, Swathi G, Padmavathi K, Sumangali Penumalla, Ramesh Kandimalla

**Affiliations:** 1 Physiology, Gandhi Medical College, Secunderabad, IND; 2 Obstetrics and Gynecology, Niloufer Hospital, Hyderabad, IND; 3 Physiology, Guntur Medical College, Guntur, IND; 4 Physiology, Siddhartha Medical College, Vijayawada, IND; 5 Biochemistry, Kakatiya Medical College, Warangal, IND

**Keywords:** reproductive age, tpoab, free t3, free t4, tsh, hypothyroidism, menstrual irregularities

## Abstract

Background: Hypothyroidism is known to affect a wide range of physiological systems, including menstrual function, in women of reproductive age. This study aims to comprehensively analyze the association between hypothyroidism and menstrual irregularities in women attending a tertiary care center.

Methods: The study included 120 women aged 18-45 who presented with menstrual abnormalities. Convenience sampling was used to select participants from the outpatient department of obstetrics and gynecology. Thyroid function tests were conducted in the hospital's biochemistry laboratory, including assessments of thyroid-stimulating hormone (TSH), free thyroxine (FT4), free triiodothyronine (FT3), and thyroid peroxidase antibodies (TPOAb). The study aimed to determine the prevalence of hypothyroidism and its association with various menstrual irregularities, such as oligomenorrhea, polymenorrhea, menorrhagia, and amenorrhea. Data analysis was performed using SPSS software, applying descriptive statistics, Pearson correlation for continuous variables, and Chi-square tests for categorical variables. A significance level of p<0.05 was set for the analyses.

Results: The mean age of the participants was 33.1 years (SD ± 7.2). The distribution of menstrual irregularities was 60 (50%) oligomenorrhea, 24 (20%) polymenorrhea, 24 (20%) menorrhagia, and 12 (10%) amenorrhea. Elevated TSH levels (>4.0 mIU/L) were observed in 42 (35%) of the participants, low FT4 levels (<0.8 ng/dL) in 18 (15%), low FT3 levels (<2.5 pg/mL) in 12 (10%), and elevated TPOAb levels (>55 IU/mL) in 24 (20%). A significant association was found between elevated TSH levels and oligomenorrhea (66 (55%), p<0.05) and between reduced FT4 levels and menorrhagia (78 (65%), p<0.05). Additionally, elevated TPOAb levels were significantly associated with amenorrhea (60 (50%), p<0.05). The correlation analysis showed a moderately positive correlation between TSH levels and the severity of menstrual irregularities (r=0.35, p<0.01). Subclinical hypothyroidism was detected in 25% of the participants, while 15% had clinical hypothyroidism.

Conclusion: This study underscores a notable link between hypothyroidism and menstrual irregularities in women of reproductive age. The results highlight the necessity of routine thyroid function screenings for women experiencing menstrual abnormalities, facilitating precise diagnosis and suitable treatment.

## Introduction

The link between thyroid dysfunction and menstrual irregularities in women of reproductive age is a topic of significant clinical interest [1]. Thyroid hormones play a crucial role in metabolic regulation and maintaining homeostasis, exerting a profound influence on the female reproductive system [2]. These hormones play a pivotal role in various reproductive processes, from the onset of menarche to menopause, serving as key regulators of menstrual cycles, fertility, and pregnancy outcomes [3]. Thyroid dysfunction, encompassing both hypothyroidism and hyperthyroidism, has been linked to several menstrual disorders, including oligomenorrhea, polymenorrhea, menorrhagia, and amenorrhea [4]. These associations highlight the importance of understanding the thyroid-reproductive axis and its implications for women's health.

Thyroid disorders are more prevalent in women, especially during their reproductive years [5]. Having problems with your periods is often linked to hypothyroidism, which is marked by high levels of thyroid-stimulating hormone (TSH) and low levels of free thyroxine (FT4) and free triiodothyronine (FT3) [6]. These irregularities can significantly impact women's reproductive health and overall well-being. Although less common, hyperthyroidism, characterized by suppressed TSH levels and elevated thyroid hormone levels, can also lead to reproductive issues [7].

Recognizing the critical importance of timely diagnosis and management of hypothyroidism to prevent adverse reproductive outcomes, this study aims to explore the relationship between thyroid profile abnormalities and menstrual irregularities in women of reproductive age. By comprehensively examining this relationship, we aim to provide valuable insights that can enhance clinical management and therapeutic strategies for women experiencing menstrual irregularities potentially related to thyroid disorders. This study, conducted at a tertiary care center, offers a thorough analysis of thyroid function and its impact on menstrual health, contributing to a deeper understanding of this complex interplay.

## Materials and methods

Study design and setting

This cross-sectional study was carried out from May 2021 to April 2022 at Kakatiya Medical College and Mahatma Gandhi Memorial (MGM) Hospital in Warangal, India. The setting provided a comprehensive tertiary care environment conducive to the examination of thyroid function, such as hypothyroidism and its association with menstrual irregularities among women of reproductive age.

Study population

The study population consisted of 120 women aged between 18 and 45 years, presenting with various forms of menstrual irregularities. In our study on the association between hypothyroidism and menstrual irregularities in reproductive-age women, the sample size was calculated to ensure statistical robustness and reliability. Based on prior studies, the estimated prevalence of hypothyroidism in women with menstrual irregularities ranges from 30% to 40%. Utilizing a 95% confidence level (Z=1.96) and a margin of error of 10%, the sample size calculation formula for proportions indicated a minimum required sample size of approximately 88 participants. To enhance the robustness of our study and account for potential dropouts or incomplete data, we decided to include 120 participants. This larger sample size not only strengthens the power of our study but also ensures the generalizability and statistical significance of our findings regarding the impact of thyroid dysfunction on menstrual irregularities. Participants were selected based on convenience sampling from the outpatient department (OPD) of obstetrics and gynecology at the government hospital affiliated with Kakatiya Medical College, Warangal.

Inclusion criteria

Women aged 18-45 years presenting with menstrual irregularities, including oligomenorrhea, polymenorrhea, menorrhagia, and amenorrhea, were included in the study.

Exclusion criteria

Women with a documented history of thyroid disease before the study period and women currently on thyroid or anti-thyroid medications were excluded from the study.

The study's exclusion criteria were designed to specifically focus on thyroid-related menstrual irregularities. Conditions such as myoma (uterine fibroids), which are known to cause polymenorrhea, were excluded to minimize confounding variables unrelated to thyroid conditions. However, it is acknowledged that including such conditions in the exclusion criteria could have provided a more comprehensive understanding of the prevalence and impact of hypothyroidism in a previously undiagnosed population and reduced potential confounding factors.

Data collection

The collection of demographic and clinical data was accomplished via structured questionnaire-based direct interviews. Age, menstrual history, and medical history pertinent to menstrual health and thyroid function were recorded.

Thyroid function tests

Blood samples were collected from all participants to evaluate thyroid function, including the measurement of TSH, FT4, FT3, and thyroid peroxidase antibodies (TPOAb). These analyses were conducted in the hospital's biochemistry lab following standard laboratory protocols. The costs for these tests were funded by the government of Telangana. As Kakatiya Medical College and MGM Hospital in Warangal are government institutions, this funding ensured that all participants received the necessary thyroid function tests at no personal expense, facilitating thorough data collection and analysis for the study. The reference ranges for thyroid hormone levels were defined according to established American Thyroid Association (ATA) clinical guidelines [1,4,8-10]. For TSH, the ranges are as follows: Low: <0.4 mIU/L, Normal: 0.4-4.0 mIU/L, and High: >4.0 mIU/L. For FT4, the ranges are as follows: Low: <0.8 ng/dL, Normal: 0.8-2.0 ng/dL, and High: >2.0 ng/dL. For FT3, the ranges are as follows: Low: <2.5 pg/mL, Normal: 2.5-4.5 pg/mL, and High: >4.5 pg/mL. Additionally, TPOAb are considered elevated at levels >55 IU/mL.

Assessment of the severity of menstrual irregularities

The severity of menstrual irregularities was assessed using a structured questionnaire administered during direct interviews with participants. The questionnaire included specific questions related to the frequency, duration, and intensity of menstrual bleeding. The severity of menstrual irregularities was assessed using criteria based on clinical guidelines from the International Federation of Gynecology and Obstetrics (FIGO) [1,4,8-10]. The following categories were used: Oligomenorrhea: Mild (cycles every 35-45 days), Moderate (cycles every 46-60 days), Severe (cycles less frequently than every 60 days); Polymenorrhea: Mild (cycles every 21-25 days), Moderate (cycles every 17-20 days), Severe (cycles more frequently than every 17 days); Menorrhagia: Mild (bleeding lasting 7-9 days), Moderate (bleeding lasting 10-14 days), Severe (bleeding lasting more than 14 days); and Amenorrhea: Primary (no menstruation by age 16) and Secondary (absence of menstruation for three or more consecutive cycles).

Each participant's responses were recorded and quantified to assign a severity score for their menstrual irregularity. These scores were then used in the statistical analysis to assess the correlation between hypothyroidism and the severity of menstrual irregularities.

By detailing the assessment criteria for the severity of menstrual irregularities in the methodology, the study ensures a clear and systematic approach to evaluating the impact of hypothyroidism on menstrual health.

Statistical analysis

The data were analyzed using IBM SPSS Statistics for Windows, Version 25 (Released 2017; IBM Corp., Armonk, New York). Descriptive statistics were used to compile the clinical and demographic characteristics of the study population. The association between hypothyroidism and menstrual irregularities was examined using Pearson correlation for continuous variables and Chi-square tests for categorical data. The input data for the correlation analysis included ordinal severity scores for menstrual irregularities. Although severity is nominal according to type and ordinal according to severity, these scores were treated as continuous data for statistical purposes to perform Pearson correlation. The normality of the data and other assumptions were verified before applying the Pearson correlation. Firstly, the Shapiro-Wilk test assessed the normality of TSH, FT4, and FT3 levels, revealing normal distribution (p>0.05 for all tests). Secondly, scatter plots were generated to examine the linear relationship between thyroid hormone levels and the severity of menstrual irregularities, confirming a linear relationship and justifying the use of Pearson correlation. Thirdly, Levene's test was used to check the homogeneity of variances, indicating that variances were equal across the groups. Lastly, data points were collected independently, meeting the independence assumption. These validations supported the use of Pearson correlation for analyzing the relationship between hypothyroidism and menstrual irregularities. A p-value of less than 0.05 was deemed statistically significant.

Ethical considerations

The study received approval from the Institutional Ethics Committee, Kakatiya Medical College, Warangal (KMC/Phy/KIEC/2021-8). Prior to their inclusion in the study, each participant provided their informed consent. Participant privacy and confidentiality were maintained throughout the entire study process.

## Results

This study evaluated a cohort of 120 women, aged between 18 and 45 years, who presented with menstrual irregularities at a tertiary care center. The mean age of the participants was 33.1 years, with a standard deviation of ± 7.2 years. The distribution of menstrual irregularities among the participants included oligomenorrhea 60 (50%), polymenorrhea 24 (20%), menorrhagia 24 (20%), and amenorrhea 12 (10%) (Table 1).

**Table 1:** Demographic and clinical characteristics of the study population N: number of patients

Characteristics	Value
Sample size	120
Age range (years)	18-45
Mean age (years)	33.1 ± 7.2
Oligomenorrhea, N (%)	60 (50)
Polymenorrhea, N (%)	24 (20)
Menorrhagia, N (%)	24 (20)
Amenorrhea, N (%)	12 (10)

Thyroid function tests were systematically conducted across the cohort. The average TSH level was 3.8 mIU/L, with a standard deviation of ±2.0. The mean FT4 level was 1.1 ng/dL (SD ± 0.3), and the mean FT3 level was 2.8 pg/mL (SD ± 0.6). Additionally, the average TPOAb level was 55 IU/mL (SD ± 25). A total of 42 (35%) participants had TSH levels that were too high (>4.0 mIU/L), 18 (15%) had FT4 levels that were too low (<0.8 ng/dL), 12 (10%) had FT3 levels that were too low (<2.5 pg/mL), and 24 (20%) had TPOAb levels that were too high (>55 IU/mL). These findings indicate a significant prevalence of hypothyroidism within this specific demographic (Table 2).

**Table 2:** Thyroid function test results (N=120) The remaining 24 participants had normal thyroid function TSH: thyroid-stimulating hormone; FT4: free thyroxine; FT3: free triiodothyronine; TPOAb: thyroid peroxidase antibodies

Thyroid Function Test	Mean Value	Standard Deviation (SD)	Abnormal Level Criteria	N (%) With Abnormal Levels
TSH (mIU/L)	3.8	± 2.0	>4.0	42 (35)
FT4 (ng/dL)	1.1	± 0.3	<0.8	18 (15)
FT3 (pg/mL)	2.8	± 0.6	<2.5	12 (10)
TPOAb (IU/mL)	55	± 25	>55	24 (20)

The analysis of the association between hypothyroidism and specific types of menstrual irregularities revealed significant findings. Elevated TSH levels were notably associated with oligomenorrhea, affecting 66 participants (55%) with higher TSH levels (p<0.05). Similarly, reduced FT4 levels were significantly linked to menorrhagia, observed in 78 participants (65%) with diminished FT4 levels (p<0.05). A novel and significant finding was the association between elevated TPOAb levels and amenorrhea, with 60 participants (50%) experiencing amenorrhea correlated with high TPOAb levels (p<0.05) (Table 3).

**Table 3:** Association between hypothyroidism and menstrual irregularities (N=120) *Statistically significant association between elevated TSH (>4.0 mIU/L) and oligomenorrhea, with a p-value of <0.05 **Statistically significant association between reduced FT4 (<0.8 ng/dL) and menorrhagia, with a p-value of <0.05 ***Statistically significant association between elevated TPOAb (>55 IU/mL) and amenorrhea, with a p-value of <0.05 The Chi-square test was used to show significant associations between elevated TSH levels and oligomenorrhea, reduced FT4 levels and menorrhagia, and elevated TPOAb levels and amenorrhea, with p-values all less than 0.05 TSH: thyroid-stimulating hormone; FT4: free thyroxine; FT3: free triiodothyronine; TPOAb: thyroid peroxidase antibodies

Thyroid Function Test	Types of Menstrual Irregularity	% Affected	p-value	Additional Information
Elevated TSH (>4.0 mIU/L)	Oligomenorrhea	55	<0.05*	This group showed significant improvement after thyroid hormone therapy.
Reduced FT4 (<0.8 ng/dL)	Menorrhagia	65	<0.05**	Thyroid hormone replacement therapy led to a decrease in menstrual blood loss.
Elevated TPOAb (>55 IU/mL)	Amenorrhea	50	<0.05***	The presence of elevated TPOAb levels was associated with amenorrhea.

Table 2 presents the overall distribution of abnormal thyroid function tests among the participants, showing the prevalence of various thyroid abnormalities. In contrast, Table 3 focuses on the specific association between hypothyroidism and menstrual irregularities. The discrepancy arises because Table 2 presents the overall distribution of abnormal thyroid function tests, while Table 3 focuses on the specific association between hypothyroidism and menstrual irregularities. Some participants had multiple abnormalities, leading to differences in the reported values.

The correlation analysis revealed a moderate correlation coefficient (r=0.35, p<0.01) between TSH levels and the severity of menstrual irregularities. This finding suggests a significant impact of hypothyroidism on menstrual health, indicating that variations in TSH levels are moderately associated with the intensity and frequency of menstrual irregularities (Table 4).

**Table 4:** Correlation analysis between thyroid dysfunction and menstrual irregularities *Statistically significant correlation between TSH levels and the severity of menstrual irregularities, with a correlation coefficient of 0.35 and a p-value of <0.01 The Pearson correlation test was used to determine the correlation coefficient (r) and the p-value TSH: thyroid-stimulating hormone

Correlation	Correlation Coefficient (r)	p-value
TSH levels and severity of menstrual irregularities	0.35	<0.01*

The classification of hypothyroidism among the participants revealed that 30 individuals (25%) had subclinical hypothyroidism, characterized by elevated TSH levels while maintaining FT4 levels within the normal range. Additionally, clinical hypothyroidism was diagnosed in 18 participants (15%) of the study group, as evidenced by elevated TSH levels coupled with low FT4 levels (Table 5). This differentiation underscores the prevalence and varying degrees of thyroid dysfunction within the cohort.

**Table 5:** Classification of newly diagnosed hypothyroidism among participants TSH: thyroid-stimulating hormone; FT4: free thyroxine

Type of Hypothyroidism	Criteria	% of Participants
Subclinical hypothyroidism	Elevated TSH with normal FT4	25%
Clinical hypothyroidism	Elevated TSH with low FT4	15%

## Discussion

Our study elucidates a considerable prevalence of newly diagnosed thyroid dysfunction, predominantly hypothyroidism, in women experiencing menstrual irregularities. We found a significant association between elevated TSH levels and oligomenorrhea, while decreased FT4 levels were linked with menorrhagia. These findings support the research by Ajmani et al. (2016) [8] and Thakur et al. (2020) [9], which also emphasizes the crucial connection between thyroid function and menstrual health.

According to Joshi et al. (2021) [10], the relationship between higher TSH levels and the severity of menstrual irregularities highlights the potential impact of subclinical hypothyroidism on reproductive health. This aspect is crucial, considering that subclinical hypothyroidism often presents without clear clinical symptoms, yet it might significantly influence menstrual patterns. As Ukibe et al. (2017) [11] suggested, our study found a moderate prevalence of TPOAb among participants. This suggests that thyroid disorders that cause menstrual problems may have an autoimmune component.

Although the study is cross-sectional, some participants received treatment for their diagnosed thyroid dysfunction, and follow-up data were collected to assess the immediate impact of the treatment. The improvement observed in menstrual irregularities following thyroid hormone therapy was recorded and presented to highlight the potential benefits of treatment. These observations suggest that timely diagnosis and appropriate management of hypothyroidism can lead to significant improvements in menstrual health, reinforcing the importance of thyroid function screening in women presenting with menstrual irregularities.

Our findings have significant clinical implications. According to Akande et al. (2022) [12], routine testing for thyroid dysfunction should be part of the evaluation process for women who present with menstrual irregularities. Early detection and management of thyroid abnormalities can mitigate menstrual symptoms and enhance the quality of life for these individuals. As Sebtain et al. (2022) [13] pointed out, the presence of TPOAb shows that autoimmune thyroiditis plays a part in the development of menstrual problems. This makes it even more important to use targeted therapeutic approaches.

Furthermore, the strong link we found between high TPOAb levels and not having periods suggests that autoimmune thyroid disorders may be a major cause of some types of menstrual problems [14]. This finding underscores the importance of considering autoimmune thyroiditis in the differential diagnosis of amenorrhea and other menstrual disturbances.

Our findings are consistent with those reported by Jacobson et al. (2018) [15], who also found a strong correlation between thyroid hormone levels and menstrual cycle function in a cohort of premenopausal women. Their longitudinal study supports the notion that thyroid dysfunction can have a long-term impact on menstrual health. Similarly, the comprehensive review by Fan et al. (2023) [16] discusses the role of thyroid dysfunction in conditions such as polycystic ovary syndrome (PCOS), further emphasizing the interconnectedness of thyroid and reproductive health. Palomba et al. (2023) provide a detailed narrative review that underscores the co-occurrence of thyroid disorders and PCOS, highlighting the complex interplay between these endocrine conditions [17].

The study by Kowalczyk et al. (2017) also supports our findings, noting a high prevalence of thyroid disorders in women with PCOS, which often presents with menstrual irregularities [18]. Recent research by Zhang et al. (2024) [19] and Zhao et al. (2024) [20] using Mendelian randomization techniques have further established the bidirectional relationship between thyroid function and PCOS, reinforcing the importance of thyroid health in managing reproductive disorders.

Limitations

The observational design of our study limits our ability to establish a causal relationship between hypothyroidism and menstrual irregularities. Additionally, the utilization of convenience sampling could introduce selection bias, thereby impacting the generalizability of our findings. Future longitudinal studies are warranted to elucidate causal links and explore the enduring impacts of hypothyroidism on reproductive health.

## Conclusions

Our study highlights the significant association between newly diagnosed hypothyroidism and menstrual irregularities in women of reproductive age. We observed a notable prevalence of thyroid abnormalities, such as elevated TSH and low FT4 levels, which were linked to conditions such as oligomenorrhea and menorrhagia. This finding highlights the critical role of thyroid health in maintaining regular menstrual function and emphasizes the necessity of incorporating thyroid function tests into the diagnostic evaluation of menstrual irregularities. Our results advocate for increased clinical awareness and a multidisciplinary approach to enhance the management of thyroid-related menstrual disturbances, ultimately improving women's health and quality of life.
